# Is aging raw cattle urine efficient for sampling *Anopheles arabiensis *Patton?

**DOI:** 10.1186/1471-2334-10-172

**Published:** 2010-06-15

**Authors:** Aneth M Mahande, Beda J Mwang'onde, Shandala Msangi, Epiphania Kimaro, Ladslaus L Mnyone, Humphrey D Mazigo, Michael J Mahande, Eliningaya J Kweka

**Affiliations:** 1 Tropical Pesticides Research Institute, Mabogini Field Station, Moshi, Tanzania; 2 Tropical Pesticides Research Institute, Division of Livestock and Human Disease vector control, Mosquito Section, P.O. Box 3024, Arusha, Tanzania; 3 Pest Management Centre, Sokoine University of Agriculture, P.O. Box 3110, Morogoro, Tanzania; 4 Laboratory of Entomology, Wageningen University & Research Centre, P.O. Box 8031, 6700 EH, Wageningen, The Netherlands; 5 Department of Medical Parasitology and Entomology, Weill-Bugando University College of Health Sciences, P.O. Box 1464, Mwanza, Tanzania; 6 KCM College of Tumaini University, P.O. Box 2240, Moshi, Tanzania

## Abstract

**Background:**

To ensure sustainable routine surveillance of mosquito vectors, simple, effective and ethically acceptable tools are required. As a part of that, we evaluated the efficiency of resting boxes baited with fresh and aging cattle urine for indoor and outdoor sampling of *An. arabiensis *in the lower Moshi rice irrigation schemes.

**Methods:**

A cattle urine treatment and re-treatment schedule was used, including a box with a piece of cloth re-treated with urine daily, and once after 3 and 7 day. Resting box with piece of black cloth not treated with urine was used as a control. Each treatment was made in pair for indoor and outdoor sampling. A 4 by 4 Latin square design was used to achieve equal rotation of each of the four treatments across the experimental houses. Sampling was done over a period of 6 months, once per week.

**Results:**

A total of 7871 mosquitoes were collected throughout the study period. 49.8% of the mosquitoes were collected from resting box treated with urine daily; 21.6% and 20.0% were from boxes treated 3 and 7 days respectively. Only 8.6% were from untreated resting box (control). The proportion collected indoors was ~2 folds greater than the outdoor. Of all mosquitoes, 12.3% were unfed, 4.1% full fed, 34.2% semi-gravid and 49.4% gravid.

**Conclusion:**

Fresh and decaying cattle urine odour baited resting boxes offer an alternative tool for sampling particularly semi-gravid and gravid *An. arabiensis*. Evaluation in low density seasons of *An. arabiensis *in different ecological settings remains necessary. This sampling method may be standardized for replacing human landing catch.

## Background

*Anopheles gambiae *complex contains the most efficient malaria vectors globally. Such vectors include *Anopheles gambiae *s.s and An. *arabiensis *[1,2]. *Anopheles arabiensis *has been associated with malaria transmission in semi arid, arid and high altitude areas [3,4]. This species may be zoophilic and/or exophilic in nature and sometimes preferring to feed on animals (especially bovines) relative to humans [5,6]. Indeed, in most of the behavioural studies, *An. arabiensis *have shown higher preference for domestic bovines as compared to other domestic animals [5-10].

Different mosquito sampling techniques are in use such as human landing catch which is highly facing ethical barriers, complex odour baited traps which needs trained staffs and CDC light traps which needs recharged power system to operate for long ; however most of them have a limited use thus new techniques are needed to complement or replace the existing ones. There is a high interest in developing other tools due to ethical concerns against human landing catch (HLC) which has been used as a gold standard [11-13]. Odour baited traps are receiving keen attention as they do not involve human as a subject and most of the time they require minimum supervision and operations during the night. Attractive odours have been shown to increase trap efficiency as female mosquitoes mainly use odour in locating their hosts [11,14]. Urine from different animals has successfully been used as attractants for different insects [15]. Cattle urine has for long time been proven as a good source of attraction for tsetse flies [15,16]. Indeed, incorporation of host odour, artificial or synthetic (cattle urine or sebum, chemical compounds) in the traps has improved the control of tsetse flies. Horse's urine has also proved a strong attraction towards tabanids [17]. Recently a study done in lower Moshi, Tanzania suggested an existing potential for using fresh cattle urine as an attractant in sampling adult *An. arabiensis *mosquitoes [11]. However, that study did not determine the duration at which urine could remain efficient as an attractant after application to the resting boxes, and effect of the aging of urine on its attraction. This study was therefore aimed at responding to such questions. Three stages of decaying cattle urine were evaluated at lower Moshi rice irrigation schemes, an area ought to have 95% of the anopheline population formed by *An. arabiensi*s [18].

## Methods

### Study area

Lower Moshi rice irrigation schemes are located in Kilimanjaro region, northern Tanzania (37°20' E, 3°21'S and 800 m above sea level). The area is hyperendemic for *Plasmodium falciparum *malaria. The area receives seasonal rainfall which mainly occurs in March-May. These rains account for about 70% of the annual total of 800 mm precipitation at Moshi Town (10-15 km north of the study area). It also receives rains during October to December. In between the two rain seasons is a hot dry season than normally occur from January-February, and cool dry season during June-September. The study was conducted between June to December; 2008. The main activity in the irrigated land is paddy cultivation, which is done throughout the year. This provides suitable aquatic environment and breeding sites thus mosquito population density remains high throughout the year. *Anopheles arabiensis *and Culicine species are most predominant mosquito species in the study area [18]. In addition to paddy farming also people practice animal and poultry husbandry.

### Urine collection and use in experiments

The urine was collected by a washing basin from a female Zebu cow (*Bos indicus*) at Mabogini village. Urine collection was done by the cattle owner and field assistants. To avoid biasness of the results, urine was always collected from the same cow. The black cotton materials were soaked into fresh urine before the experiments. Soaked clothing materials were wrapped on the inside of resting boxes as described by Kweka *et al*. [11]. Experiments were conducted in a 4 by 4 Latin square design with four different treatments. The treatments included box with cloth material treated with urine daily (Trap A); box with cloth material treated once in every 3 days (Trap B); box with cloth treated once in every 7 days (Trap C); and box with cloth material not treated with urine but only wetted with distilled water only (control) (Trap D). Each trap was prepared in pairs for indoor and outdoor mosquito collections.

### Mosquito sampling

Four sites known to produce large number of mosquitoes were selected. The distance from one site to the other was 1 km, and a total of 8 houses were selected, 2 per site. Houses were used for both outdoor and indoor sampling rotations. The indoor and outdoor samplings were done in comparable houses. The resting boxes were placed in the evening at 18:00 h and sampling of mosquitoes was done at 6:30 to 7:00 h. Traps were rotated among the four houses such that each trap had a chance of being tested in each of the houses. Mosquitoes were sampled once a week for the period of six months.

### Mosquito collection and processing

Mosquitoes from the resting boxes were collected using hand aspirator, put into paper cups and provided with 10% sugar solution before they were transported to the laboratory and killed using chloroform. Mosquitoes were counted and identified morphologically using taxonomic keys as described by Gillies and Coetzee [2]. All *An. gambiae *obtained were regarded as *An. arabiensis *as Ijumba *et al*. [18] revealed that the species form >95% of the mosquito population in the study area. The abdominal conditions of collected mosquitoes were graded as unfed, fed, semi-gravid and gravid as per standard procedures described in the WHO entomological manual [19].

### Data entry and analysis

Data were double entered in MS-access database for validation. Analysis was performed using the SPSS programme (Version 17.0 for Windows, SPSS Inc., Chicago, IL). Variables which affected mosquito density were analysed using the univariate generalized linear model. The analyzed factors included days, position of the sampling box (indoor/outdoor), treatment and house location. Analysis of the difference between indoor and outdoor sampling of each treatment was done using paired sample t-test. Descriptive statistic was used to establish the means and 95% confidence intervals of each treatment for the outdoor and indoor catches. The comparisons were considered statistically different at *p *< 0.05.

## Results

**Figure 1:**
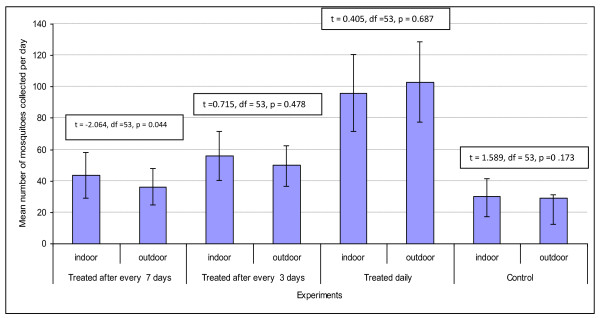
**Variation of mosquitoes abundance in each sampling box according to treatment days**.

The total number of mosquitoes collected throughout the study period was 7871, out of which 3920 (49.8%) were from box treated with cow urine daily, 1700 (21.6%), box re-treated after every 3 days, 1574 (20%) box re-treated after every 7 days and 677 (8.6%) non-treated box (control). In overall, the mean number of mosquitoes collected varied with urine re-treatment schedule (DF = 2, F = 21.879, *p *< 0.0001), daily re-treatment being more effective than re-treatment after every 3 and 7 days. Generalized linear model revealed that the proportion of mosquitoes collected in each of treatment and control was dependent on day of collection, house position and whether collection was done indoor or outdoor (df = 3, F = 45.3, *p *= 0.021). 2941 and 4830 mosquitoes were collected indoors and outdoors respectively. For box trap treated daily, the proportion of *An. gambiae *s.l collected outdoors was slightly higher but not different from that of indoors (*p *= 0.18). But, for box traps re-treated every 3 and 7 days, greater proportion of *An. gambiae *s.l was collected indoors than outdoors (*p *< 0.001, Figure 1). All *An. gambiae s.l *were presumed to be *An. arabiensis *basing on the finding by Ijumba *et al*. [18] who reported >95% of *An. gambiae *s.l to be *An. arabiensis *in the area where this study was conducted. Of the collected *An. gambiae *s.l, 968 (12.3%) were unfed, 323 (4.1%) freshly fed, 2692 (34.2%) semi-gravid and 3888 (49.4%) gravid.

## Discussion

This study tested aging urine to indicate the possibility of its use for sampling *An. arabiensis*. Kweka *et al*. [11] proved the usefulness of fresh cattle urine for sampling *An. arabiensis *under field settings. Fresh urine has also been used for field collection of tsetse flies [20-22]. Information on the length of time at which cattle urine remains attractive after application remains necessary and will assist in determining re-treatment rates and improvement.

Although not as much as with fresh urine, the present study has demonstrated collection of high proportion of mosquitoes using traps re-treated 3 and 7 days after initial application. Out of 7871 mosquitoes which were collected, 21.6% and 20% were from traps re-treated with urine every 3 and 7 days respectively. 8.6% was collected from traps not treated with urine. This signifies that cattle urine could still be useful for collecting mosquitoes several days post its initial application. In studies where urine was evaluated against tsetse no flies were collected in the traps with aging urine [17,21,22].

The proportion of mosquitoes collected indoor was ~2 folds greater than that collected outdoor. *Anopheles arabiensis *is 25 to 50 percent exophilic in nature depending on the locality [6], thus we would expected to have more of them collected outside than inside houses. This might be partially due to absence of wind blows indoors hence causing the boxes to be wet for long time with concentrated odour than those boxes outdoor which may be affected by wind blow. Despite the difference in indoor and outdoor catches, it is still worthy saying that resting boxes can be used for indoor and outdoor sampling of *An. arabiensis*. This suggests the potential of incorporating synthetic or biological insecticides in cattle urine baited traps (lure and kill) to target outdoor or indoor resting mosquitoes. However, for the outdoor targeting, box traps will require a water proof casing to ensure their use during rains. In addition to lure and kill, using urine odours may increase the efficiency of the existing tools without using humans as baits. This will ensure determination of malaria vectors and parasites dynamics using simple and ethically acceptable tools rather than human landing catch. Study done in northern Tanzania showed that CDC light trap were by 22.64% more efficient than clay pots [23]. Coupling pots with cattle urine odour will probably increase their efficiency thus offering additional traditional sampling tools as effective as CDC light trap.

The resting box traps captured all range of mosquitoes with different abdominal status, unfed (12.3%), freshly fed (4.1%), semi-gravid (34.2%) and gravid (49.4%). However, a large proportion was made of semi-gravid and gravid mosquitoes. Similarly, in the study where pots were used, many sampled mosquitoes were semi-gravid and gravid [23]. After feeding, mosquitoes are badly looking for conducive places to rest, digest blood and lay eggs. Conditions within the resting boxes were probably good enough thus many fed mosquitoes remained. It could be that, almost equal numbers of unfed mosquitoes were attracted by the odour but escaped to continue scavenging for blood source hence lower density of unfed *An. gambiae *s.l.

Cheap but effective sampling tools are obviously required for sustainable use in the malaria endemic regions where coincidentally >85% of the people are poor and live in rural areas. The present study used resting boxes and black cotton cloth material which can be obtained locally and at relatively low cost. The boxes are portable and of small sized and can be placed in any place without disturbing house owners. For routine surveillance not many areas would always afford to use tools like Magnetic traps with synthetic odours although such tools have been used successfully [24-26]. Other sampling tool such as odour baited entry trap have shown to be effective and protective to human participants but needs to have several machines, tents and a security [7,25].

## Conclusion

Fresh and decaying/aging cattle urine can be used to odour bait resting boxes thus increasing their efficiency in sampling *An. arabiensis*, more so for semi-gravid and gravid mosquitoes. However, evaluation in low density seasons of *An. arabiensis *in different ecological settings will yield more information to aid in the rational use of resting boxes as sampling tools. Later odour baited resting boxes can be standardized to replace human landing catch as a sampling tool for *An. arabiensis *and other malaria vector species.

## Conflict of interests

The authors declare that they have no competing interests.

## Authors' contributions

AMM and EJK conceived and designed the study, analysed data and prepared the first draft and edited this manuscript. SM, HDM, EEK, MJM, BJM and LLM collected data. LLM edited the manuscript. All authors read and approved this final version.

## Pre-publication history

The pre-publication history for this paper can be accessed here:

http://www.biomedcentral.com/1471-2334/10/172/prepub
